# Knowledge, attitude and practice towards malnutrition and micronutrient deficiency among male and female farmers in Ethiopia

**DOI:** 10.1186/s40795-023-00791-0

**Published:** 2023-11-14

**Authors:** Girma Gezimu Gebre, Derebe Ermias Chefebo, Deribe Kaske Kacharo

**Affiliations:** 1 Faculty of Environment, Gender and Development Studies, Hawassa University, Hawassa, Ethiopia; 2 The Japan Society for the Promotion of Science (JSPS) Postdoctoral Research Fellowship Program, Ritsumeikan University, Kyoto, 603-8577 Japan; 3 Department of Agricultural Economics, College of Agriculture and Natural Resource, Werabe University, Werabe, Ethiopia

**Keywords:** Gender, Malnutrition, Micronutrient deficiency, Ethiopia

## Abstract

**Background:**

Despite a large body of literature on the nexus between knowledge, attitude and practice towards nutrition and gender, this nexus is likely to vary and is not clear in many societies, such as Ethiopia.

**Objectives:**

The study aimed to analyze the level of gender-based knowledge, attitude, and practice towards malnutrition and micronutrient deficiency using primary data collected from two regional states in Ethiopia.

**Methods:**

Qualitative and quantitative data collection approaches were used. Qualitative data were analyzed using a narrative and content approach. Quantitative data were analyzed using descriptive statistics.

**Results:**

Results indicate that female are generally more adept than male at identifying the symptoms of malnutrition. However, concerning vitamin A and iodine food types and its deficiency, male respondents had relatively better knowledge and consumption practice than female. Results show that there is very little awareness about biofortified and fortified foods. When we rate respondents, male had a relatively better understanding about fortified foods than their female counterparts.

**Conclusion:**

Findings can support development agents working to improve nutrition in Ethiopia to focus on improving community knowledge and perception of biofortified and fortified foods to improve diet quality through increased micronutrient intake. The majority of the respondents were aware of the importance of consuming micronutrient rich foods and had a positive attitude towards them. However, there is still a gap in practice. It may therefore require more targeted campaigns to increase the ability of community members to adopt best practices while reducing barriers to consumption of nutritious diet.

**Supplementary Information:**

The online version contains supplementary material available at 10.1186/s40795-023-00791-0.

## Introduction

Global statistics show that more than two billion individuals, or one in three people globally, suffering from hidden hunger. Hidden hunger is the presence of multiple micronutrient deficiencies such as iron, iodine and vitamin A; which can occur without a deficit in energy intake as a result of consuming an energy-dense, but nutrient-poor diet [1]. When we are deficient in a specific micronutrient, say vitamin A, iron, and iodine, our brain does not get or trigger the same signal as our body's needs for more food. This form of hunger is known as hidden hunger or micronutrient deficiency [1].

Malnutrition is an abnormal physiological condition, typically due to deficiencies or excesses in nutrient intake, or imbalances of essential nutrients in the body. Malnutrition, in all its forms (under nutrition or over nutrition) affects almost every country in the world, leading to serious public health risks and incurring high economic costs [2]. Micronutrient deficiencies, particularly those in folic acid, iodine, iron, and vitamin A, have a long-lasting impact on growth and development and are thus a national priority [3]. Improvements in nutrition will significantly contribute to reducing poverty and achieving the health, education, and employment goals outlined in the United Nations [2]. Nutrition stimulates economic growth, which improves the physical productivity and mental health of the labor force. The Inter-Agency Standing Committee (IASC) noted that girls and boys—and men and women—have different nutritional needs at different life stages. They face different risks and challenges in accessing sufficient nutrition. Gender inequality exacerbates food insecurity, malnutrition and poverty in humanitarian crises [4]. The socially constructed gender roles of men and women interact with their biological roles to affect the nutrition status of the entire family and of each gender [5, 6]. All gender and age groups have the right to equal access to nutrition services and the foods they need to live a healthy life [4]. Women are the main food producers, yet they are disproportionally affected by hunger and malnutrition. Evidence suggests that when women have more control over how and how much time they spend feeding their children, and when women have better access to healthcare, the prevalence of undernutrition decreases (Royal Tropical Institute [KIT] and Netherlands Development Organization [7–9]. The nutritional status of women (before, during, and after pregnancy) is intimately linked with the nutritional status of their children [5, 7, 8, 10]. However, despite global efforts to address under-nutrition among women and children, the prevalence of under-nutrition remains high [7, 8].

Even though it was at a slower pace, Ethiopia has made progress in the reduction of child stunting and maternal undernutrition in the last two decades. For example, the prevalence of stunting has decreased from 58% in 2000 to 37% in 2019, depicting an average decline of 1.25 percentage points a year [11]. On July 15^th^, 2015, the Government of Ethiopia made a declaration to end child malnutrition by 2030, reaffirming its commitment to nutrition as a foundation for economic development. Accelerating progress towards this goal, set out in the *Seqota Declaration*, will require coordinated multisectoral efforts to increase nutrition, smart investments in infrastructure and technology, behavior change, and empowering communities to innovate and identify localized solutions to address malnutrition [12]. Eliminating undernutrition in Ethiopia would prevent losses of 8–11% per year from the gross national product [3].

Women are among the most at-risk for poor nutrition, particularly in Ethiopia, where economic and social disparities tend to be greater [13]. Even when food is available at home, women tend to be malnourished because of their gender status. Women shoulder “triple roles”, including their reproductive, productive, and social (community) responsibilities [14–16]. These roles place a significant burden on women, increasing their risk of malnutrition [13]. A study by [17] in Ethiopia indicated that women have a lower decision-making authority than men within households regarding decisions on the proportion of produced food consumed at home. Sociocultural and traditional norms often result in women consuming smaller amounts of food or foods with less nutritional diversity, as well as prioritizing the more nutritious food items for men. Effectively understanding sociocultural structures and gender dynamics has served to strengthen results from interventions for improved nutrition practices or enforcing nutrition programmes with education on rights and advocacy skills [18].

Despite a large body of literature on the nexus between nutrition and gender, this nexus is likely to vary and is not clear in many societies, such as Ethiopia. Hence, this paper contributes to literature on the nexus between nutrition and gender using primary data collected from Ethiopia. The objectives of this study were (i) to analyze the level of male and female farmers’ knowledge, attitude, and practice towards malnutrition and micronutrient (e.g. vitamin A, iron, and iodine) deficiency; and (ii) to assess the status of consumption of micronutrient-rich foods among farming households in the study area.

## Materials and methods

### Study area and data

**Fig. 1:**
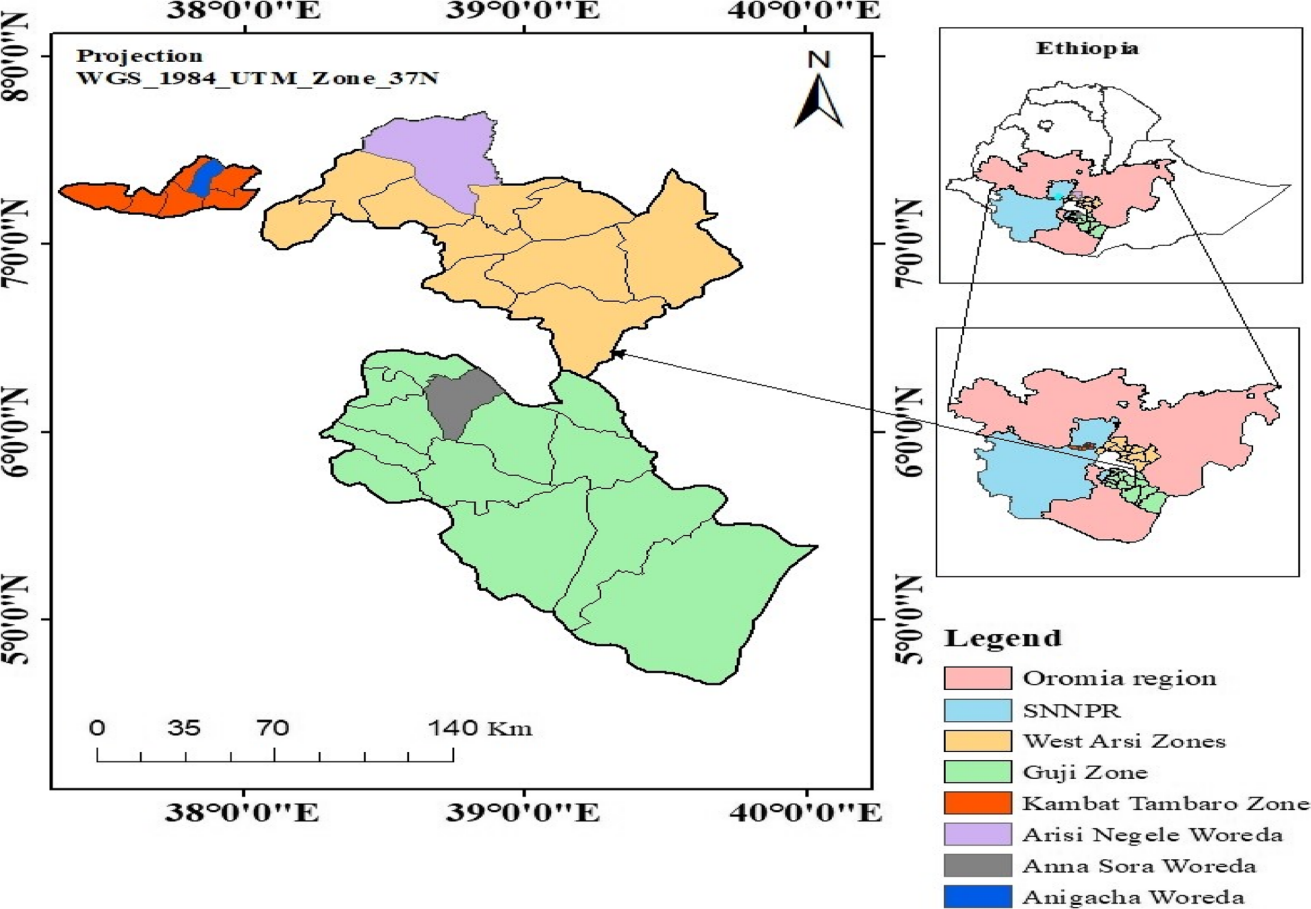
Location of the study area, Source: Authors

The study is based on primary data collected in December 2021 in three woredas (Angecha woreda in the South Nation Nationalities and People Region (SNNPR), Arsi Negelle, and Anna Sora woredas in the Oromia region) of Ethiopia. Angacha woredas are located in Kembata, Tembaro zone of the SNNPR in Ethiopia. Anna Sora and Arsi Negelle woredas are located in the Guji and West Arsi zones of the Oromia region in Ethiopia, respectively (Fig. 1). The study applied a mixed methods approach consisting of qualitative data collection through focus group discussion (FGD) and key informant interview (KII) and quantitative data collection through concisely formed questionnaires. The study used a convergence research design through which the qualitative and quantitative data are collected and analyzed during a similar timeframe. The KII and FGD served as the basis for including local context on key KAP issues found in the study. These were done by letting the FGD participants and key informants explain the reasons behind the KAP findings of this study.

### Sample and data collection methods

#### Sampling techniques

Detailed FGD guides and questions were developed in close coordination with the quantitative data collectors. The discussions were conducted by the researcher to verify the self-reported information by the household respondents and to provide an in-depth understanding of the reported nutrition KAP. Discussions were held in the local language, which was a translation from English by professional local language interpreters. Illustrations related to the topics were used to stimulate discussion and engage the participants. On average, FGD consisted of six participants each. In order to avoid mutual influence in the responses, only one member of each household was randomly selected. A total of 21 groups, with a total of 126 participants, were formed separately in accordance with age, gender, and characteristics to allow the participants to speak freely. The farmer's training center was used as a venue for conducting FGD. Each discussion lasted no more than one hour. The FGDs were conducted with the following groups (Table 1).

**Table 1:** Qualitative (FGD and KII) sample

Locations				Number of FGD by gender category and KIIs					
Region	Zone	Woreda	Kebelle	Male group	Female group	Mixed group	Average number of participants in group	KII	Total number of participants in FGD and KII
Oromia	Guji	Anna Sora	AbaboKobo	1	1	1	6	2	20
			Raya Boda	1	2	1	6	3	27
	West Arsi	Arsi Negelle	Gubete Arjo	1	1	1	6	1	19
			Turge Gallo	1	1	1	6	2	20
SNNPR	Kambata Tambaro	Angecha	Kerkicho	2	2	-	6	3	27
			Sino Funamura	2	2	-	6	3	27
Total	3	3	8	8	9	4	21 *6 = 126	14	140

A KII guide was carefully developed based on broader lines of inquiry to further explore gender and nutrition issues. The KIIs consisted of woreda agricultural extension coordinators, agricultural development agents working at kebele level, and health extension service providers. In each kebele, 1–3, key informants were invited to talk to the qualitative team to provid detailed information concerning household knowledge, attitudes, and practices towards malnutrition, vitamin "A", iron, and iodine deficiencies (Table 1). Moreover, the interview focused on issues related to gender and nutrition in the community. The interviews took place at their homes or offices separately in order to create a safe space for them to talk about this sensitive topic. Explicit consent to interview each person was collected and data from these interviews containing sensitive issues on gender and nutrition-related issues is kept under the strictest confidence. Data collected from KII were transcribed by the lead researcher who clearly understand the local language.

A multi-stage sampling procedure was applied to collect quantitative data. First, woreda was selected, followed by kebele, household, and individual respondent in each household. At the household level, the sample size of 311 respondents (185 males and 126 females) was calculated using Fisher’s formula [19, 20]. Accordingly, from each household, one respondent was randomly selected, totaling 50 to 54 interviews per kebele (Table 2). The quantitative data collectors consisted of six locally based enumerators and two nutrition-sensitive research supervisors. Each interview took one hour per person on average. The interviews were conducted by trained enumerators at a farmer's home using a mobile data collection tool called Kobo Collect. Kobo Collect is a smart phone application applicable for field survey data collection. Of course, there are different smart phone applications useful for field survey data collection, such as Survey Solution. But Kobo Collect is easy to use in any rural area with no access to internet service. We used this tool for very large survey data collection, including the USAID. This tool has applied in several studies been including our previous published works (e.g. [16, 17]).

**Table 2:** Quantitative sample respondents

Region	Zone	Woreda^**a**^	Kebele^**b**^	Number of respondents		
				Male	Female	Total
Oromia	Guji	Anna sora	Ababo Kobo	37	14	51
			Raya Boda	35	15	50
	West Arsi	Arsi Negelle	Gubete Arjo	20	31	51
			Turge Gallo	30	21	51
SNNPR	Kambata Tambaro	Angecha	Kerkicho	32	22	54
			Sino Funamura	31	23	54
Total	3	3	8	185	126	311

^a^Woreda (District) is the third level of the administrative division of Ethiopia – after zone and the regional states

^b^Kebele is the lowest admirative unit or peasant association in Ethiopia. It is the smallest administrative unit of Ethiopia, contained within a woreda in Ethiopia

Training of enumerators took place over four days, where three days occurred in a classroom setting, and one day in a selected community for pre-testing. The training schedule included the basics of a KAP study overall, interviewing methodology by enumerators, review of questionnaires, translation from English (orally) to local languages, and pre-testing of questionnaires in the field (see questionnaire attached as online Supplementary material).

The pre-testing involved reviewing the qualitative and quantitative instruments to ensure the questions were clear, understandable, relevant to the intended topics, effective in providing useful information and, more importantly, to avoid redundant or unnecessary questions. Pre-testing also allowed enumerators and qualitative researchers to verify the correct local words and phrases of some of the complex ideas to which the study team wanted to gain insights on nutrition and gender. Pre-testing was conducted immediately prior to data collection in Kofele woreda of West Arsi zone, close to the study area.

Regarding components of the questionnaire used for quantitative data collection, the first part of the questionnaire comprised of demographic details such as age, marital status, sex of the household head, education level, household size, and farming experience. The latter part consisted of nutrition-related KAP questions. In total, the questionnaire is comprised of 41 KAP questions that encompass 16 knowledge questions, 7 practice questions, and 18 attitude-related questions.

The household interviews were conducted in the local language (Kambaatissa and Afan Oromo). Also, the questionnaire on the data collection platform, Kobo Collect, was in both Kambaatissa and Oromifa. This enables the enumerators to be intimately familiar with the questionnaire and be comfortable during interview time.

The participation of all research subjects in this study was voluntary and collected in a written form. Participants were informed before an interview or discussion took place about the purpose and were given the opportunity to refuse upon understanding the purpose. No exercise of undue inducement or any other form of coercion to participate in the study was permitted or accepted.

### Data analysis

Qualitative data were analyzed using a narrative and content approach. Quantitative data were analyzed using descriptive statistics such as frequency distribution, mean comparison, percentages, and chi-square test. Moreover, a Likert scale was used to establish the respondent’s attitude towards malnutrition. SPSS software was used to analyze quantitative data.

## Results

### Respondents socio-demographic characteristics

Table 3 presents the socio-demographic characteristics of the surveyed respondents. Male and female respondents account for 59.5% and 40.5% of the total (311) surveyed respondents, respectively. The study focused on men and women to get their opinion as well as gauge their nutrition knowledge, attitude, and practices. The same knowledge would facilitate an in-depth understanding of how men and women get involved and participate in decisions pertaining to the household. The overall average age of sampled respondents was 38 years old*.* The mean age of male and female respondents was 39.3 and 35.1, respectively. As a result of the T-test, the mean difference between males and females was statistically significant at 1% (*p* = 0.001). This indicates that, male respondents become more aware of malnutrition as their age increases, contrary to female respondents. This implied that higher level of understanding and deeper experience in household nutritional issues come with age.

**Table 3:** Socio-demographic characteristics of the respondent in the household

Characteristics	Total (*n* = 311)	Male (*n* = 185)	Female (*n* = 126)
Age of the respondent in years	38	39	35
Mean	37.562	39.259	35.071
SD	10.939	12.028	8.561
T-test	-3.369		
*p*-value	0.001***		
Respondent relationship in the household			
Male head	64	95	17
Female head	6	0.0	16
Housewife	27	0.5	67
Other	3	5.0	0.8
Pearson chi^2^	225.365		
*p*-value	0.000***		
Education level of respondent			
No formal education	13	8	22
Primary education (1^st^ – 8^th^ grade)	54	50	59
Secondary education (9^th^ – 12^th^)	29	35	19
Higher education (> 12 grade)	4	7	0.0
Pearson chi^2^	32.431		
*p*-value	0.000***		
Marital status of the respondent			
Married	93	96	88
Single	3	3	2
Divorced	1	0.0	2
Widowed	4	1	8
Pearson chi^2^	14.798		
*p*-value	0.002**		
Total number respondent household members	7	7	7
Mean	7.128	7.183	7.047
SD	2.610	2.742	2.412
T-test	-0.451		
*p*-value	0.652		
Number of male household members above 15 years	2	2	2
Number of female household members above 15 years	2	2	2
Number of children below or equal to 15 years	3	3	3
Amount of years respondent has been farming	21	23	19
Mean	20.864	22.448	18.539
SD	10.156	10.949	8.377
T-test	-3.387		
*p*-value	0.001***		

Categorical variables are presented as percentages

** and *** denote level of significance at 1% and 5%, respectively

The household heads with longer farming experience are supposed to have better understanding of malnutrition of the household than the household heads with shorter farming experience. The mean year of farming experience of male and female respondents was 22.448 and 18.539, respectively. As a result of T-test, the mean difference between males and females was statistically significant at 1% (*p* = 0.001). This indicates that, male respondents become more aware of malnutrition as their year of farming increases, contrary to female respondents. This implied that, household head with longer farming experience were to be more knowledgeable and practicable regarding household malnutrition.

More than half of the respondents (54%) had only primary level education. The importance of the level of education in gender equality is also underscored by the World Bank, which notes that the low levels of education, especially among women, represent a very serious constraint on development in most of the sub-Sahara African countries, Ethiopia not being exceptional. At the individual level, for example, education is perceived to be the ultimate liberator, which empowers a person to make personal and social choices [21]. The World Bank argues that education is also perceived to be the ultimate equalizer, particularly in promoting greater gender equity for women. Education is very important for farmers to understand malnutrition. Farmers who have high formal education are expected to be aware of malnutrition earlier than uneducated; because farmers with higher education levels were able to get information from different sources. The study results also revealed that the education level of the household head has a positive relationship and is statistically significant (chi^2^ = 32.431, *p* = 0.000) at 1% of level (Table 3).

Nearly all (92%) respondents were married and the total average household size was 7, which is higher than the national average of 5. The mean household size for male and female-headed households was 7.2 and 7.0 respectively. The statistical analysis also, revealed that there is no significant difference (0.652) in the mean household size between male and female household head.

### Knowledge and attitude towards malnutrition

#### Knowledge about malnutrition

The results of the study on Table 4 show that, the majority of male (96%) and female (92%) can recognize if someone in their household is malnourished. The results of chi-square analysis indicate that recognition of malnutrition in the household has positive relationship but statistically not significant (x^2^ = 2.5013; *p* = 0.114) (Table 4). About 85% of respondents know that lack of energy or weakness, are the main symptoms of being malnourished, while 58% and 84% of respondents know that weakness of the body's immune system and loss of weight/thinness, respectively, are the main symptoms of being malnourished in their respective households. FGD and KII participants also stated that, weakness, less immunity, chest pain, and headache were the most common symptoms of malnutrition at household and community level in the study area.

**Table 4:** Knowledge and attitude towards malnutrition

	All (%)	Male (%)	Female (%)
**Respondent knowledge about occurrence of malnutrition in the household**			
Can recognize if someone in the household is malnourished?	95	96	92
Pearson chi^2^	2.501		
*p*-value	0.114		
Symptoms of being malnourished			
Lack of energy/weakness:	85	87	81
Weakness of the immune system	58	56	61
Loss of weight/thinness	84	84	83
Don`t know about symptom of malnutrition	0.6	1	0.0
Reasons why individuals in the household do not consume balanced diet			
Not having enough money to buy food	87	90	83
Food is not available/difficulty obtaining food	71	72	70
Inappropriate dietary choices	38	35	44
Other health problems such as mental conditions	24	22	27
Intra-household distribution problem	2	21	25
Don’t know	2	2	2
How to prevent malnutrition in the household			
Eating fortified food	41	43	40
Foods enriched with micronutrients	72	76	67
Avoiding monotonous dish or eating variety food	35	35	36
Fair distribution of food among family members	49	50	48
Awareness creation to make right food choice	77	78	75
Improving income to afford for nutritious food	75	71	81
Grow diversified vegetables and fruits at home garden	2	1	2
Don’t know	1	1	2
**Respondents attitude towards malnutrition in the household**			
How likely do you think your household may have malnourished members?			
Not likely	74	75	73
Not sure	3	4	3
Likely	21	21	22
Most likely	0.6	0.0	2
Pearson chi^2^	3.105		
*p*-value	0.376		
How serious is malnutrition for your household members health?			
Not serious	57	57	57
Not sure	8	6	10
Serious	24	25	24
Very Serious	11	11	10
Pearson chi^2^	1.166		
*p*-value	0.761		

It was found that 87% of study participants had insufficient money to buy food, and 71% were unable to access multiple food groups. A majority of respondents (72%) suggested that eating foods enriched with micronutrients such as iron and vitamin "A" would prevent malnutrition problems in their household. Around 77% of respondents said that raising awareness among household members about making healthy food choices would help prevent malnutrition, while 75% and 49% said that increasing household income to afford nutritious food in the market and distributing food fairly among family members in the household would help prevent malnutrition (Table 4).

#### Attitude towards malnutrition

A Likert scale was used to establish the respondent’s attitude towards malnutrition. Nearly three-quarters (74%) of the total respondents were *not likely* to think that their household may have malnourished members, while 22% of the respondents were *likely* to think that there would be malnourished members in their household. The results of chi-square test indicate that attitude towards malnutrition in the household has positive relationship but statistically not significant (x^2^ = 3.1058; *p* = 0.376). More than half of the respondents (57%) did not think malnutrition was *a serious* problem for household members' health and only 11% of respondents thought malnutrition was a *very serious* issue in their household. According to the results of the chi-square test, attitudes towards malnutrition in the household have a positive relationship, but the relationship is not statistically significant (× 2 = 3.1058; *p* = 0.376) (Table 4).

### Consumption of iron-rich foods and iron -deficiency”

#### Knowledge about iron-rich foods and iron deficiency

Table 5 presents respondent knowledge, attitude, and practice about iron-rich foods in the household. Regarding the sources of iron-rich foods, 78% of the respondents chose red meat as their major source of iron-rich foods, while 66%, 59%, and 34% chose teff (injera*- a flat spongy Ethiopian bread mostly made of fermented teff flou*r), butter, and pumpkin, respectively. 9% of the respondents had no knowledge about the sources of iron-rich food. A majority of the respondents (93%) knew about the benefits of eating iron-rich foods.

**Table 5:** Consumption of iron rich foods in the household

	Total (%)	Male (%)	Female (%)	Chi-square	*p*-value
**Respondent knowledge about Iron deficiency and iron-rich foods**					
Heard about iron-deficiency anaemia					
Yes	87	87	88	0.057	0.810
No	11	12	9		
Don`t know about iron-deficiency anaemia	2	1	3		
Examples of iron rich foods					
Red meat	78	79	78	4.101	0.043
Teff (injera)	66	65	66		
Pumpkin	34	32	36		
Butter	59	58	60		
Don`t know	9	7	11		
Know benefits of eating iron rich foods?	93	93	92	0.090	0.764
Problems observed by not consuming iron rich foods					
Less energy/weakness	83	85	81	0.824	0.364
Headache	50	48	52	0.547	0.459
Paleness	69	67	72	0.948	0.330
Stomach pain	31	29	33	0.396	0.529
Vomiting	29	31	25	1.075	0.300
Do not know	8	7	8	0.030	0.861
**Household practice on taking Iron rich foods**					
Consumed iron rich foods, yesterday, during the day or night	53	53	60	4.101	0.043
**Attitude towards Iron rich foods deficiency**					
Likelihood of a household member being iron deficient?					
Not likely	40	44	35	3.752	0.290
Not sure	10	8	13		
Likely	41	40	44		
More likely	9	9	9		
Seriousness of not consuming foods rich in iron?					
Not serious	12	12	13	4.358	0.225
Not sure	5	4	6		
Serious	59	56	64		
Very serious	23	28	17		
How good do you think it is for your household to prepare meals with iron-rich foods?					
Not good	0.3	0.0	1	2.583	0.460
Not sure	1	0.5	2		
Good	31	30	32		
Very good	68	70	66		
Difficulty in preparing meals with iron rich foods?					
Not difficult	18	16	22	3.503	0.174
Somewhat difficult	48	52	42		
Very difficult	34	32	36		
Confidence to prepare meals with iron rich foods?					
Not confident	16	15	17	2.219	0.528
Less confident	57	61	52		
Confident	24	22	28		
More confident	3	3	3		
Taste acceptability of iron-rich foods?					
Dislike	5	5	6	2.636	0.268
Neutral	7	9	4		
Like	88	91	90		

Body weakness, paleness, and headache were the most common symptoms of inadequate intake of iron-rich foods reported by the respondents. The majority of the respondents (88%) reported that they had heard about iron-deficiency anemia. Moreover, FGD and KII participants reported that there are incidences of anemia in the community.

#### Attitude towards consumption of iron-rich foods and iron-deficiency

More than half of the respondents (60%) think that it is *a serious* problem when their household members do not eat iron-rich foods. Nearly 7 out of 10 respondents (68%) think it *is good* to prepare meals with iron-rich foods such as red meat, chicken, liver, and dark green vegetables. Approximately 34% of respondents said it is extremely difficult for their households to prepare meals rich in iron, while less than 18% said it is not difficult. Almost three-quarters (73%) of the respondents are not confident in preparing meals with iron-rich foods, indicating a perceived ability to prepare iron-rich foods is a major barrier, yet most of them (88%) *like* the taste of iron-rich foods such as red meat, liver, injera, and chicken.

About 4 out of every 10 respondents (40%) think that it is *not likely* to have iron-deficient household members. Those with less than 10% think it would be *most likely* to have a household member who is iron deficient.

#### Consumption practices of iron-rich foods

About half (53%) of survey respondents consumed iron-rich foods in the last 24 h prior to the survey. The most commonly consumed iron-rich foods reported by the surveyed respondents were *Teff* (injera), legumes (mixed beans, baked beans, lentils, chickpeas), and dark leafy green vegetables.

### Consumption of vitamin “A” rich foods

#### Knowledge about vitamin A-rich foods

Table 6 presents respondents’ knowledge, attitude, and practice regarding vitamin A-rich food consumption in the household. Most surveyed respondents (89%) had heard about human health problems such as night blindness or inability to see in dim light in their community. About 83% had heard about Vitamin "A" deficiency or diseases caused by not consuming vitamin A-rich foods such as eggs, carrots, cheese, orange-fleshed sweet potatoes (OFSP), milk, or yoghurt (full cream dairy). In open-ended questions, respondents were asked to list vitamin "A" rich foods they are consuming in their households. Their responses were summarized as butter, milk, carrot, OFSP, and don't know for the purpose of presentation. Accordingly, most of the respondents (88%) reported carrots as a major source of vitamin "A", while 75%, 58%, and 39% noted milk, butter, and OFSP, respectively, as the major sources of vitamin "A". About 6% of the respondents were not aware of the major sources of vitamin "A" rich foods. Respondents were also asked about their knowledge about the benefits of eating vitamin "A" rich foods such as biofortified foods (e.g., orange-fleshed sweet potatoes) and fortified foods (e.g., fortified oil, wheat flour, and iodized salt). The majority of them, 90%, knew about the benefits of eating vitamin "A" rich foods.

**Table 6:** Consumption of Vitamin “A” rich foods in the households

	Total (%)	Male (%)	Female (%)	Chi-square	*p*-value
**Respondent knowledge about Vitamin “A” rich foods deficiency**					
Awareness of vitamin A deficiency related symptoms	89	89	88		
Aware of Vitamin “A” deficiency	83	86	78		
Knowledge of example Vitamin “A” rich foods?					
Butter	58	63	52	3.806	0.051
Milk	75	76	72		
Carrot	88	91	85		
OFSP	39	41	36		
Do not know	6	4	10		
Awareness on benefits of Vitamin “A” rich foods	90	93	86	4.400	0.036
Awareness on benefits of orange-fleshed sweet potato (OFSP)	40	39	41	0.173	0.678
Aware of benefits of consuming fortified foods?	71	72	70	0.246	0.620
**Consumption of Vitamin “A” rich foods**					
Consumed Vitamin “A” rich foods yesterday	83	87	77		
Access of Vit A rich food by household members?					
Children	22	21	23		
The mother	3	2	3		
The fathers	19	18	20		
All family members have equal access	57	59	54		
Produce or buy orange fleshed sweet potato?	58	56	60	0.517	0.472
Buy fortified edible oil/ wheat flour?	78	79	77	0.271	0.602
**Attitude towards Vitamin “A” rich foods deficiency**					
How likely do you think any of your household member lacks vitamin “A” in his/her body?					
Not likely	38	37	40	5.943	0.114
Not sure	12	9	15		
Likely	46	51	40		
More likely	4	3	6		
How serious do you think a lack of vitamin A is?					
Not serious	23	22	24	1.273	0.736
Not sure	7	6	8		
Serious	52	51	52		
Very serious	19	21	16		
How good do you think it is to prepare meals with vitamin-A-rich foods?					
Not good	0.6	0.5	0.8	3.165	0.367
Not sure	1	0.5	2		
Good	27	25	30		
Very good	71	74	67		
How difficult is it for your household to prepare meals with vitamin- A-rich foods?					
Not difficult	22	17	28	7.895	0.019
Somewhat difficult	50	56	40		
Very difficult	29	27	32		
How confident do you feel in preparing meals with vitamin-A-rich foods?					
Not confident	14	12	17	2.998	0.392
Less confident	54	52	50		
Confident	29	28	31		
More confident	2	3	2		
How much do you like the taste of Vitamin “A” rich foods?					
Dislike	3	2	5		
Neutral	2	2	2		
Like	95	96	93		

#### Attitude towards vitamin A-rich foods

The respondents were asked how *likely* they thought it was that any of their household members lacked vitamin "A". Thus, nearly half (46%) believe that it is *likely* Over half (52%) of respondents said vitamin A deficiency is serious. Seventy-one percent of respondents feel confident that they can prepare meals containing vitamin-A-rich foods. About 50% of respondents thought it was somewhat difficult to prepare foods rich in Vitamin A. About 54% of respondents were feel less confident in preparing meals with vitamin-A-rich foods in the household. The majority (95%) of the respondents *like* the taste of vitamin-A-rich foods (Table 6). These results indicate that respondents perceive a positive attitude towards eating vitamin A-rich foods.

#### Consumption practices of vitamin A-rich foods

A majority (83%) of the respondents consumed vitamin "A" rich foods in the past 24 h prior to this survey. More than half (57%) of the respondents stated that all family members have equal access to vitamin A-rich foods. About 58% of the respondent’s household uses biofortified and fortified foods, such as orange-fleshed sweet potatoes. Whereas, 79% of their households use fortified foods such as fortified edible oil or wheat flour (Table 6).

### Consumption of iodized salt

#### Knowledge about consumption of iodized salt

Survey results indicate that a majority of respondents (69%) had information about human health problems related to iodine deficiency, such as goiter, apathy, and muscle weakness (Table 7). All of the respondents stated that their household uses salt to cook meals.

**Table 7:** Consumption of iodine or iodized salt in the household

	Total (%)	Male (%)	Female (%)	Chi-square	*p*-value
**Respondent knowledge about iodine deficiency**					
Have you heard about iodine deficiency or problems related to not eating iodized salt (e.g. goitre, apathy, and muscle weakness)?					
Yes	69	72	66	1.452	0.484
No	23	21	26		
Don`t know	8	8	8		
Do you or any one in your household know how can iodine deficiency be prevented?					
Yes	58	57	59	0.307	0.858
No	25	26	24		
Don`t know	17	17	17		
**Respondent household practice on taking iodized salt**					
Did you or household use salt to cook the main meal eaten by members of your family yesterday/ in last week?	100	100	100		
What kind of salt did you use?					
Iodized	39	38	41	1.394	0.498
Not iodized	445	47	40		
Don`t know	16	15	18		
**Respondent attitude towards use of iodized salt**					
How likely do you think your household lacks iodized salt at home?					
Not likely	31	30	33	1.628	0.653
Not sure	16	17	14		
Likely	32	30	35		
More likely	21	23	18		
How serious do you think not using iodized salt in the body is?					
Not serious	17	16	19	1.659	0.646
Not sure	33	33	33		
Serious	42	42	43		
Very serious	8	9	6		
How good do you or your household think it is to prepare meals with iodized salt?					
Not good	2	3	2	1.067	0.785
Not sure	21	21	20		
Good	47	49	44		
Very good	31	29	34		
How difficult is it for your household to buy and use iodized salt?					
Not difficult	57	55	60	1.945	0.378
Somewhat difficult	29	29	29		
Very difficult	14	16	10		

#### Attitude towards consumption of iodized salt

Results show that 32% of respondents think "it is *likely*" and 31% think "it is *not likely*" to lack iodized salt at home (Table 7). About (42%) think that the lack of iodized salt is a *serious* issue. About half (47%) were *confident* that they could prepare meals with iodized salt, and a slight majority (57%) stated that it was *easy* for their household to buy and use iodized salt. Only 14% said it was *very difficult* for their household to buy and use iodized salt.

#### Consumption practices of iodized salt

All surveyed respondents responded that they use salt to cook meals, with 39% using iodized salt and 45% using non-iodized salt (Table 7). All FGD and KII participants stated that the local community usually consumes non-iodized salt and on very rare occasions they consume packed and iodized salt. Some of the reasons given were lack of awareness about the existence of so-called iodized salt; they only knew about common or regular salt. Affordability (high price compared to the normal one) was also stated as a reason for not using iodized salt; even though they were aware of its existence, it was not available for purchase in local markets and shops. The results confirm that there is a knowledge and practice gap in the consumption of iodized salt in the study area.

## Discussion

This study was intended to assess the level of KAP among male and female farmers towards malnutrition and micronutrient deficiency; and the status of consumption of micronutrient-rich foods. The study findings revealed that, in general, the majority of respondents had poor knowledge, attitudes, and practice towards reducing malnutrition. This is in line with studies that revealed in all communities, regardless of differences in socioeconomic class and educational level, knowledge, attitudes, and practices about vitamin A were low [22, 23]. However, this result contradicts the study`s finding that the majority of caregivers had knowledge of important baby and young child feeding practices [24].

39% and 71% of the respondents knew about the benefits of eating orange-fleshed sweet potatoes and fortified foods, respectively. Moreover, there were no such knowledge differences in the two group' about micronutrients. However, FGD and KII participants reflect contrary to the study findings regarding community knowledge of biofortified and fortified foods. They believe that, non-fortified oil is consumed more than fortified oil, as fortified oil was sold at a higher price in the study area. Also, there is a lack of knowledge on fortified oils' health benefits and types of nutrients added in the fortified oil. In SNNPR (Angecha woreda), fortified wheat flour is not known to the community. Farmers in Angecha woreda used to go to the "grinding mill house" and prepare the wheat flour locally. Even when they buy wheat flour from the market, it is the same and there is no access to fortified wheat flour. When they buy the processed and packed wheat flour, they don’t care whether it is fortified or not. Unlike survey participants, FGD and KII participants were reported that, Orange-fleshed sweet potato (OFSP) was not being produced in their community and people were not aware of the health benefits of eating OFSP. Even more educated professionals, such as development agents, health extension workers, and kebele coordinators who participated in KII, are not aware of the orange-fleshed sweet potato. In general, consumption of regular sweet potatoes is also quite rare in the study area. This misalignment between FGD and KII participants and survey respondents may be attributed to the misunderstanding of what fortified and biofortified foods are when asked in the survey, and the closed nature of a questionnaire does not allow enumerators to verify with follow-up questions. Similar findings were reported by WFP [25] in the KAP study on maternal nutrition, infant and young child feeding, sanitation and hygiene, and sexual and reproductive health, including obstetric fistula, in Chemba District, Sofala in Mozambique. In the [25] study, all FGD participants stated that they are unable to identify fortified foods, so they cannot distinguish fortified and non-fortified foods in the market, and they cannot make an informed choice when it comes to buying micronutrient supplements or fortified products. They indicated that they have never been told about the existence of these products by any communication channels in the communities in Mozambique. However, approximately 70% of survey respondents in the WFP study stated that they are aware of fortified foods. Moreover, 64% of survey respondents (66% of men and 62% of women), indicated they purchase fortified foods in the market for their children, and only a mere 3% indicated they do not.

Females are generally more adept than males at identifying the symptoms of malnutrition. However, concerning vitamin A and iodine food types and its deficiency male respondents had relatively better knowledge and consumption practice than female. Compared to female, male respondents had better understanding about fortified food in the study area.

According to the findings, a lack of income (87%) was the primary cause of a lack of a balanced diet in the study locations. As reported in Table 4, the most crucial elements in preventing malnutrition are increasing sources of income so that people can purchase healthy food and raising awareness so that people may make wise food choices. Malnutrition was stated by both the FGD and KII respondents as the central problem in the study community/area. In terms of nutrition, the majority of the families under investigation continue to struggle with hunger and food insecurity in general, especially in Angecha woreda.

## Conclusion and recommendations

The aim of this study was to assess nutrition-related knowledge, attitudes, and practice among male and female farmers in the Oromia and SNNP regions of Ethiopia. The findings from this study will inform the nutrition and gender-related development project implementation with a focus on its social behavior change communication (SBC) plan. Results indicate that female are generally more adept than male at identifying the symptoms of malnutrition. However, concerning vitamin A and iodine food types and its deficiency male respondents had relatively better knowledge and consumption practice than female.

Findings also show that the benefits of biofortified and fortified foods are still largely unknown among community members. Even more educated professionals such as development agents, health extension workers, and kebele coordinators who participated in KII are not aware of biofortified foods such as orange-fleshed sweet potatoes. To supplement the government of Ethiopia's efforts to increase consumption of micronutrient rich foods such as orange-fleshed sweet potatoes, high iron pearl millet, and fortified foods such as iron fortified wheat flour, iodized salt, and vitamin A fortified oils; non-governmental organizations such as Sasakawa Africa Association need to raise awareness about the benefits of consuming biofortified and fortified foods. Therefore, awareness creation is needed for extension experts and community members on the benefits of producing and consuming biofortified and fortified foods. 

The findings show that the majority of the respondents are aware of the importance of consuming vitamin A and iron-rich foods, iodized salt, as well as production and consumption of fruits and vegetables and have a positive attitude towards them. However, there is still a gap in practice. As a result, more targeted campaigns may be required to increase community members' ability to adopt best practices while reducing barriers to consumption of nutritious diet. The current SBC strategy should change from an awareness creation campaign to a behavior change campaign, focusing on perceptions of the benefits and practices of the production and consumption of vitamin A and iron-rich foods as well as the consumption of iodized salt.

### Supplementary Information

**Additional file 1. **Household survey questionnaire.

## Data Availability

Data used in this study are confidential. Data would be available up on reasonable request from the corresponding author.
