# Supplementary material for: Knowledge, attitude and practice towards malnutrition and micronutrient deficiency among male and female farmers in Ethiopia
Source: BMC Nutr. 2023 Nov 14;9:130. doi: 10.1186/s40795-023-00791-0 (PMC10647073; doi:10.1186/s40795-023-00791-0)
Supplement: Supplementary file 1 — Additional file 1. Household survey questionnaire. [file 40795_2023_791_MOESM1_ESM.docx]

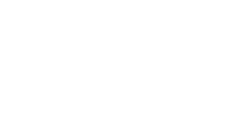

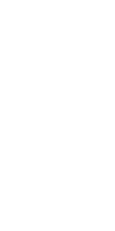

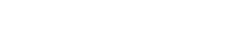
**Household Survey questionnaire**

**Consent Form**

| Good morning/afternoon, Mr/Mrs/ _________________. I am conducting research on KAP toward malnutrition and micronutrient deficiency in this area. The objective our research is to collect baseline data on nutrition knowledge, attitudes, and practice, and collect actionable insights to help development agents improve its nutrition messages for social behaviour change promotion amongst its beneficiaries in this area. You have been selected to participate in this survey study that will take about one hour. Your participation is entirely voluntary. Your answers will be completely confidential; I will not share information that identifies you with anyone. After entering the questionnaire into a data base, I will destroy all information such as your name which will link these responses to you.  Do you agree to participate in this survey/interview?  Yes ___ No ___ If yes, continue to the next question; if no, stop the interview |
| --- |

1. Socio-demographic profiles of the respondent in the household
2. Date of interview: Date _______Month____________Year______________________________
3. Interviewed by (enumerator name):_________________________________________________
4. Name of the respondent______________________________. Respondent Code____________
5. Phone Number ______________________
6. Region: A. SNNPR B. Oromia
7. **Zone**: A**.** Guji B. Negelle Aresi C. Kambata Tambaro
8. **Woreda**: A. Anna sora B. Negelle Aresi C. Angacha
9. **Kebele**: A. Raya Boda, B. Sololo Kobo C. Ababa Kobo D. Gubeta Arjo E. Kerkicho F. Sino funamura
10. Sex of the respondent A. Male B. Female
11. Age of the respondent ______________years
12. Respondent relationship /responsibility/ in the household A. Female Head B. Wife C. Husband D. Other (relatives, boy/girl or grandmother/grandfather)
13. Education Background (your highest level of education)
14. Primary Education B. Secondary Education C. Higher Education D. No education
15. Total number of respondent household members___________
16. Number of male household members above 15 years __________________
17. Number of female household members above 15 years _________________
18. Number of children below 15 years _____________
19. The amount of years respondent has had in farming _______________
20. **Occurrence or severity of Malnutrition in the household**

**Respondent knowledge about malnutrition problem**

**K.1.** Can you recognize if someone in your household is having malnourished (under/over nourished food intake)?

1. Yes. 2. No.

**K.2.** What is/are symptom/s of being malnourished?

1. Fatigue
2. Lack of energy/weakness: cannot work, study or play as normal (disability)
3. Weakness of the immune system (becomes ill easily or becomes seriously ill)
4. Loss of weight/thinness
5. Others__________________________________________________

**K.3.** What would be the reasons why people (e.g. your household members) do not get balanced nutrition/food? (Multiple answers possible)

1. Not having enough money to buy food
2. Food is not available/difficulty obtaining food
3. Inappropriate dietary choices
4. Other health problems such as mental conditions
5. Intra-household distribution problem (e.g. men or children get served first)
6. Others___________________________________________________

**K.4.** What should be done to prevent malnutrition problem in your household? (Multiple answer is possible)

a. Eating biofortified food such purified oil, wheat flour, iodized salt etc.

b. Avoiding monotonous dish or eating variety food

c. Fair distribution of food among family members in the household

d. Awareness creation to households to make right food choice

e. Improving household income to afford for nutritious food in the market

f. Grow diversified vegetables and fruits at home garden

g. others_________________________________________

Respondent Attitudes towards malnutrition related problem

**A.1**: How likely do you think your household may have undernourished members, that is they stop growing or lose weight?

1. Not likely 2. Not sure 3. Likely 4. Most likely

If Not likely: Can you tell me the reason why it is not likely? ____________________________________

**A.2**: How serious is malnutrition for your household members health?

1. Not serious 2. Not sure 3. Serious 4. Very serious

If Not Serious: Can you tell me the reason why it is not serious? __________________________________

1. Consumption of Iron rich foods in the household

Household respondent knowledge about Iron rich foods deficiency (anaemia)

**K.1:** Have you heard about iron-deficiency anaemia (e.g. extreme fatigue, weakness or eating not much, pale skin, chest pain, fast heartbeat or shortness of breath, headache, bleeding during menstruation etc.)?

1. Yes 2. No 3. Don`t know about iron deficiency you mentioned above

**K.2:** Among the listed below, what are iron rich foods? You can give more than one answer

1. Lean meat 2. teff (injera) 3. Pumpkin 4. tomatoes 5 Butter

**K.3.** Do you know about benefits of eating iron rich foods such as red meat, injera, spinach, or Iron-fortified foods?

1. Yes 2. No

**K.4:** Among the listed below, what are the common problems/ you observed by not taking iron rich foods such as liver, red meat, injera, pumpkin? You can answer to more than one)

1. less energy/weakness 2. headache 3. Paleness 4. Stomach pain 5. Vomiting

**Household practice on taking Iron rich foods**

**P.1:** Did you or any of your household member eat iron rich foods (e.g. meat, chicken, spinach, pumpkin, fish, legumes, fruits, etc..), yesterday, during the day or/and night?

1. Yes, 2. No

**P.2:** Among the food items mentioned above, who has more access in the household members?

1. Children 2. The mother 3. The fathers 4. All family members have equal access

Household respondent Attitudes towards Iron deficiency problem

**A.1**: How likely do you or your household think you or any of your household member is to be iron deficient?

1. Not likely 2. Not sure 3. Likely. 4. More likely

If Not likely: Can you tell me the reason why it is not likely? ____________________________________

**A. 2**: How serious do you think not eating foods rich in iron?

1. Not serious 2. Not sure 3. Serious. 4. Very serious

If Not Serious: Can you tell me the reason why it is not serious? __________________________________

**A.3:** How good do you think it is for you or your household to prepare meals with iron-rich foods such as red meat, injera, chicken, liver, or pumpkin?

1. Not good 2. Not sure 3. Good 4. Very good

**A.4**: How difficult is it for you or your household to prepare meals with iron-rich foods?

1. Not difficult 2. Somewhat difficult 3. Very difficult

If Not difficult: Can you tell me the reason why it is not difficult? _________________________________

**A.5:** How confident do you feel in preparing meals with Iron rich foods?

1. Not confident 2. Less confident 3. Confident 4. More confident

**A.6:** How much do you like the taste of red meat, liver, injera, chicken, pumpkin and other iron rich foods?

1. Dislike 2. Neutral 3. Like

1. Consumption of Vitamin “A” rich foods in the household

Household respondent knowledge about Vitamin “A” rich foods deficiency

**K.1:** Have you heard about human health problems such as night blindness or inability to see in dim light, dry eye), less immunity to infections, weakness etc?

1. Yes 2. No

**K.2:** Have you heard about Vitamin “A” deficiency or diseases caused by not taking foods such as egg, carrot, cheese, milk or yoghurt in your household or community?

1. Yes 2. No

**K.3:** Among the listed below, what are Vitamin “A” rich foods? You can give more than one answer

1. Butter 2. Milk 3. Lemmon 4. carrot 5. Orange

**K.4.** Do you know about benefits of eating Vitamin “A” rich foods?

1. Yes, 2. No

**Practices of taking Vitamin “A” foods in the household**

**P.1:** Did you or any of your household member eat source of Vitamin “A” foods (e.g. meat, carrot, egg, milk and milk products, vegetables, potato etc.) yesterday, during the day or/and night?

1. Yes 2. No

**P.2:** Among the food items mentioned above, who has more access in the household members?

1. Children 2. The mother 3. The fathers 4. All family members have equal access

Household respondent attitudes towards Vitamin “A” deficiency

**A.1**: How likely do you think you or any of your household member is to lack vitamin “A” in his/her body?

1. Not likely 2. Not sure 3. Likely 4. More likely

If Not likely: Can you tell me the reason why it is not likely? _______________________________

**A. 2:** How serious do you think a lack of vitamin A is?

1. Not serious 2. Not sure 3. Serious 4. Very serious

If Not serious: Can you tell me the reason why it is not serious? --------------------------------------

**A. 3:** How good do you think it is to prepare meals with vitamin-A-rich foods such as carrots, green leafy vegetables, orange fleshed sweet potato or meat for your family members?

1. Not good 2. Not sure 3. Good 4. Very good

**A.4:** How difficult is it for your household to prepare meals with vitamin- A-rich foods?

1. Not difficult 2. Somewhat difficult 3. Very difficult

If Not difficult: Can you tell me the reason why it is not difficult? ______________________________

**A.5:** How confident do you feel in preparing meals with vitamin-A-rich foods?

1. Not confident 2. Less confident 3. Confident 4. More confident

**A.6:** How much do you like the taste of Vitamin “A” rich foods such as egg, carrot, meat, milk, yoghurt etc?

1. Dislike 2. Neutral 3. Like

1. Consumption of on Iodine or iodized salt in the household

Respondent Knowledge about iodine deficiency

**K.1:** Have you heard about iodine deficiency or problems related to not eating iodized salt (e.g. feeling cold, constipation, dry skin, goiter, apathy, weight gain, muscle weakness etc)?

1. Yes 2. No 3. Don`t know

**K. 2:** Do you or any one in your household know how can iodine deficiency be prevented?

1. Yes 2. No 3. Don`t know

**Practices of use of Iodized salt in the household**

**P.1:** Did you or household use salt to cook the main meal eaten by members of your family yesterday/ in last week?

1. Yes 2. No

**P. 2:** What kind of salt did you use?

1. Iodized 2. Not iodized 3. I do not know

Attitudes towards use of iodized salt

**A.1:** How likely do you think you or your household is to lack iodized salt at home?

1. Not likely 2. No sure 3. Likely 4. More likely

If Not likely: Can you tell me the reason why it is not likely? ____________________________________

**A. 2:** How serious do you think a lack of iodine (not using iodized salt) in the body is?

1. Not serious 2. Not sure 3. Serious 4. very serious

If Not serious: Can you tell me the reason why it is not serious? -----------------------------------------------

**A. 3:** How good do you or your household think it is to prepare meals with iodized salt?

1. Not good 2. Not sure 3. Good 4. Very good

**A. 4:** How difficult is it for you or your household to buy and use iodized salt?

1. Not difficult 2. Somewhat difficult 3. Very difficult

If Not difficult: Can you tell me the reason why it is not difficult? ______________________________

**I have finished my questions. Do you have any?**

Thank you very much for your time and cooperation!


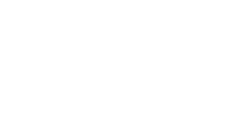

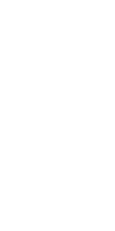

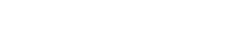


**Focus Group Discussion check lists (for men and women groups)**

**Community Members**

| Good morning/afternoon, Mr/Mrs/ _________________. I am conducting research on KAP toward malnutrition and micronutrient deficiency in this area. The objective our research is to collect baseline data on nutrition knowledge, attitudes, and practice, and collect actionable insights to help development agents improve its nutrition messages for social behaviour change promotion amongst its beneficiaries in this area. You have been selected to participate in this survey study that will take about one hour. You have been selected to participate in this FGD that will take about 1 hour. Your answers will be completely confidential; we will not share information that identifies you with anyone. After entering the questionnaire into a data base, we will destroy all information such as your name which will link these responses to you. |
| --- |

Date________________ Name of interviewer________________________

Location: Region_________________zone________________woreda_______kebele________

1. What are the major roles/activities that, women, and men in your community engage in? When and where are these roles/activities carried out? (focus on adult groups: 15-65 age groups)

- Men roles/activities
- Women roles/activities
- Girls roles/activities
- Boys roles/activities

1. What are the cultural norms surrounding women’s participation in farming within this community?
2. What are other barriers that prevent women in this community from engaging in community decision making meetings?
3. Is malnutrition (taking under or over nutritious food) a problem in this community? How and what causes it? What should be preventive measures?
4. What do you do when you see the symptoms or problems of under or over nutrition in your household or community?
5. What do you do when you see the signs of the symptoms mentioned above (ref Q#5)?
6. In your household and community what are the most common foods consumed regularly? (Grains, dairy, poultry, meat, fruits, vegetables)
7. What makes the food items discussed above commonly used? (E.g. availability, cost, taste)
8. What are the most common sickness at household and community level among the following?
   1. Children
   2. Men
   3. Women
   4. What are the causes that you anticipate?
   5. How do you treat it?
9. Are the foods mentioned above (table) easily available to the majority of the people living in this community?
10. How is the practice of consuming iodized salt in your case and among the community in general?
11. Do you know about benefits of consuming fortified foods such fortified foods such as maize flour/wheat flour, purified oil? Are these foods available in your community?
12. Do you know about benefits of consuming biofortified foods such as orange fleshed sweet potato? Are these foods available in your community?

**I have finished my questions. Do you have any question?**

**Thank you very much for your cooperation!**


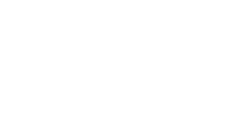

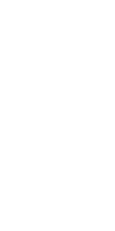

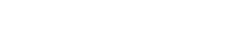
**Questions for Key Informant Interview (KII)**

| Good morning/afternoon, Mr/Mrs/ _________________. I am conducting research on KAP toward malnutrition and micronutrient deficiency in this area. The objective our research is to collect baseline data on nutrition knowledge, attitudes, and practice, and collect actionable insights to help development agents improve its nutrition messages for social behaviour change promotion amongst its beneficiaries in this area. You have been selected to participate in this survey study that will take about one hour. You have been selected to participate in this FGD that will take about 30 minutes. You are selected to participate in this KII that will take about 1 hour. Your participation is entirely voluntary. Your answers will be completely confidential; we will not share information that identifies you with anyone. After entering the questionnaire into a data base, we will destroy all information such as your name which will link these responses to you.  Do you agree to participate in this interview?  Yes ___ No ___ If yes, continue to the next question; if no, stop the interview |
| --- |

Possible candidates will be stakeholders; health bureau, extension workers, DA’s, etc.

Date________________ Name of interviewer________________________

Location: Region_________________zone________________woreda_______kebele________

**Guiding Questions**

1. Based on your experience with community engagement, can you describe gender beliefs and practices in the state that influences the level of women’s participation in household decision making related to food consumption and health practices?
2. What other barriers limits the involvement of women?
3. How best do you think women like you can be reached with information in this community? Probe for reaching women with information around farming.
4. What role do the duty bearers play with respect to household nutrition?
5. What can be done to improve a woman’s participation in deciding what the households eats?
6. I want you to rank the following in terms of who's opinion is most important for your community in terms of deciding on family food preference and consumption.
   1. The mother b. the father c. extension workers d. children e. elders (mother in laws/ father in laws)
7. In the community do you feel there are good food varieties/options for the communities?
   1. How does this affect the health situation of the community?
8. From your observation, who gets relatively good meals in the family?
   1. Why?
9. What are the most observed sickness in the community?
   1. What do you think is the cause?
   2. What measures are taken in times of sickness in most cases?
